# Thyroid Metastasis from Nonsmall Cell Lung Cancer

**DOI:** 10.1155/2013/208213

**Published:** 2013-12-19

**Authors:** Tariq Namad, Jiang Wang, Ralph Shipley, Nagla Abdel Karim

**Affiliations:** ^1^Division of Hematology and Oncology, University of Cincinnati, College of Medicine, Cincinnati, OH 45267, USA; ^2^Division of Pathology, University of Cincinnati, College of Medicine, Cincinnati, OH 45267, USA; ^3^Division of Radiology, University of Cincinnati, College of Medicine, Cincinnati, OH 45267, USA

## Abstract

*Background*. Thyroid metastases are rare. Clinically, they represent less than 4% of thyroid malignancy in clinical studies. *Aim*. To assess various presentations and therapy for patients with lung cancer metastatic in the thyroid. *Materials and Methods*. We report a case of metastatic adenocarcinoma of the lung to the thyroid. We reviewed similar reports through PubmMed search from 1997 until 2013. *Case Presentation*. A 48-year-old lady was seen in the clinic for an adenocarcinoma of left upper lobe (LUL) of the lung; she received neoadjuvant chemotherapy then LUL lobectomy. After 9 months she presented with diffuse goiter initially believed to be a solitary metastatic lesion as it was positive for adenocarcinoma of lung origin on histopathological exam with TTF-1 positivity. Unfortunately, PET scan showed additional mediastinal lymphadenopathy. *Conclusion*. The treatment strategy for metastatic thyroid disease is based on a multidisciplinary approach, where thyroidectomy would have been considered in case of a solitary metastatic involvement, but further metastatic workup is mandated to direct further systemic therapy versus palliative radiation therapy.

## 1. Background

Malignancies with intrathyroid metastases from other primary malignancies are rare. Clinically, they represent less than 4% of all thyroid malignancies in clinical studies [1]. The most common malignancies that have been reported to metastasize to the thyroid are the breast, lung, and kidney cancers [2]. Of the pulmonary malignancies metastasizing to the thyroid, adenocarcinomas are the most common type followed by squamous cell, small cell, and large cell carcinomas, respectively. The distinction between primary and secondary malignant thyroid tumors by clinical examination and imaging can be challenging [3, 4]. A history of cancer can be of help in reaching the diagnosis; however, the final confirmation by histopathology is required.

## 2. Case Report

A 48-year-old female, prior smoker, who was diagnosed with T2N1M0, Stage IIB adenocarcinoma of left upper lobe (LUL) of the lung, the EGFR, and ALK being negatives. She was treated with neoadjuvant cisplatin and pemetrexed for 3 cycles and followed by LUL lobectomy.

**Figure 1:**
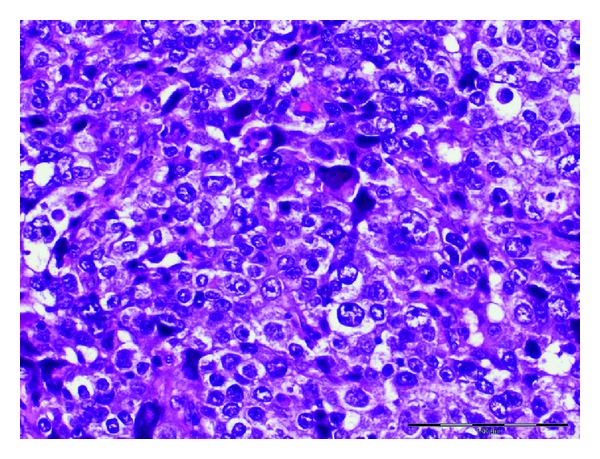
Lung lobectomy: hematoxylin and eosin stain of the pathologic specimen from left upper lobectomy showing poorly differentiated adenocarcinoma, H&E.

**Figure 2:**
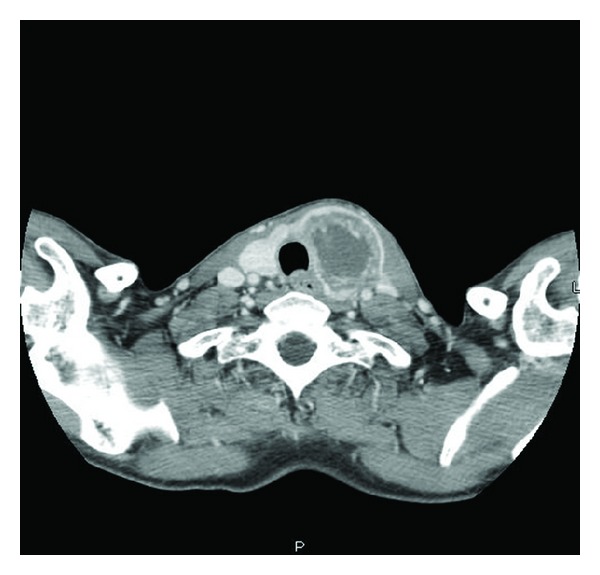
Neck CT: A reveals a large heterogeneous mass that has replaced the left lobe of the thyroid gland.

**Figure 3:**
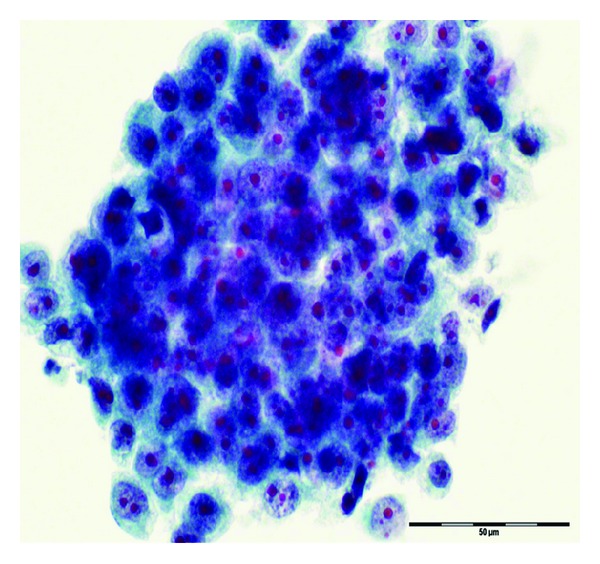
Fine needle aspiration of the thyroid mass FNA, ThinPrep shows clusters of cancer cells with large nuclei and prominent nucleoli, consistent with metastatic lung adenocarcinoma.

**Figure 4:**
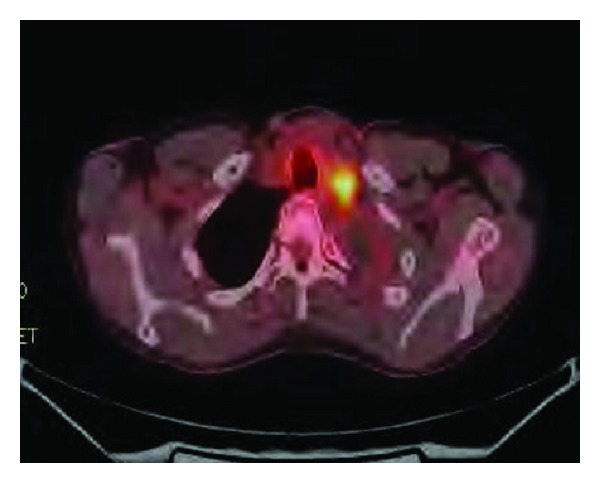
Fused fluorodeoxyglucose (FDG) positron emission tomography (PET) CT shows hypermetabolic left supraclavicular lymphadenopathy (yellow).

Histopathological examination of the tumor confirmed poorly differentiated adenocarcinoma (Figure 1). Surgical course was complicated by superior mesenteric artery (SMA) syndrome for which a gastrostomy-jejunostomy (G-J) tube was placed on and she was started on tube feeds, temporarily at that time. This had prevented her from undergoing in further adjuvant systemic therapy. After nine months of her initial presentation diagnosis, she returned with progressive fatigue, dyspnea, and dysphagia. She denied any cough, fever or weight loss. On physical exam, she was noted to have diffuse goiter, with no significant lymphadenopathy. The haemogram, renal, and liver function tests were normal. A modified barium swallow revealed a mildly delayed in swallowing with pooling to the vallecula; however, there was no esophageal compression from the thyroid mass. The ultrasound revealed multiple thyroid nodules; the largest was in the left thyroid and measured 2.4 × 2.0 cm. Neck computed tomography (CT) showed interval enlargement of a heterogeneously enhancing mass in the left thyroid lobe (Figure 2). A fine needle aspiration (FNA) revealed clusters of malignant epithelial cells. Immunohistochemistry demonstrated that these cells were positive for TTF-1 and negative for thyroglobulin, consistent with metastatic disease from lung origin (Figure 3). The patient was evaluated for thyroidectomy; nevertheless, because of the presence of positron emission tomography (PET) positive mediastinal lymph nodes that were hypermetabolic (Figure 4), she was deemed a nonsurgical candidate. Given her earlier good response to a platinum and pemetrexed combination, she was restarted on doublet systemic therapy with carboplatin and pemetrexed. She had stable disease with no response after 3 cycles and worsening symptoms, so her therapy was switched to docetaxel, and radiation therapy was consulted for palliation in case of lack of response to systemic therapy.

## 3. Discussion

Lung cancer is the leading cause of cancer death worldwide in both men and women, with an estimated 1.4 million deaths each year [5]. Common sites of lung cancer metastasis include brain, bones, adrenal glands, contralateral lung, and liver.

Metastasis to the thyroid gland is rare and occurs mainly in autopsy cases described in the literature [6]. Among the most common and known malignancies that metastasized to the thyroid are breast, lung, and kidney cancers [2]. However, the incidence of metastases to the thyroid gland is rare, comprising only 2% to 4% of all clinical cases of malignant thyroid tumors [1, 2], and most cases in the literature have been identified at autopsy [6]. The metastases to the thyroid gland occur usually through hematogenous spread [1, 4]. Although the thyroid gland is the most vascularized gland after the adrenals [1], it is rarely the site of metastases. On autopsy series, breast and lung were the most frequently observed primary cancers to metastasize to the thyroid [4, 7]. However, other clinical series have noted that the primary renal clear cell carcinoma was the most commonly associated with thyroid metastases compared to the breast and lung cancer [2–4, 8]. They were found to be up to 24% in cadaveric studies [9].

Thyroid metastases represent less than 4% of all malignant thyroid tumors. According to Nakhjavani et al. [2], the number of cases of thyroid metastases reported in the literature has increased, although this may be due to more frequent thyroid FNA or a selection bias. The peak age for thyroid metastases is in the sixth decade [1, 3, 4, 10].

Typically, the interval between the diagnosis of the primary tumor and the detection of thyroid metastasis is from one month to twenty-six years [1, 10]. In this case report, the interval was nine months after the lung cancer diagnosis. The clinical manifestations of thyroid metastases are rare as they could only be encountered on imaging studies [10]. Patients usually present with a thyroid nodule or goiter [3, 4, 7]. Patients mostly complain of the most cervical discomfort, dyspnea, dysphagia, or dysphonia [4, 10], which may also involve vocal cord or laryngeal paralysis [3, 4] and should alert the clinician, especially in a patient with a history of previous malignancy.

Biologically, the thyroid hormone balance is usually unaffected by the metastatic thyroid involvement nodules [11]. Ultrasound imaging would generally show thyroid metastatic lesions as hyperechoic masses, and cervical scanner with and without injection CT may show calcifications in the thyroid parenchyma, multinodular thyroid enlargement, or an isolated nodule. CT is also useful to assess the impact on adjacent organs, including the trachea and esophagus [12].

Thyroid scan usually shows a cold nodule [12]. A PET scan, which is usually obtained to complete the diagnostic work up, may show a hypermetabolic mass indicative of the thyroid metastasis [8, 13].

Fine needle aspiration (FNA) with ultrasound guidance is a rapid, minimally invasive, and inexpensive technique for the diagnosis of metastatic lung cancer in the thyroid gland [7]. A thyroid nodule detected in a patient with a history of recent or old remote cancer should be considered for FNA to rule out metastatic disease in the thyroid [10]. However, FNA might be noncontributory and not to guide the etiological diagnostic if insufficient cells are available to make the cell block used for immunostains. In addition, it might be difficult to distinguish anaplastic thyroid cancer from metastatic malignancies due to its poor differentiation and lack of expression of TTF-1 [1, 2, 7]. TTF-1 has been shown to be positive in primary lung adenocarcinoma and in the majority of primary thyroid cancers; therefore, clinical presentation and the use of thyroglobulin staining may be important for the final diagnosis.

A positive immunostaining for thyroglobulin suggests the diagnosis of a primary thyroid neoplasm, although a primary thyroid neoplasm may still be present with negative immunostaining [7, 8].

The treatment of thyroid metastases depends on the primary site the malignancy, the stage of the disease, and whether surgical resection would be considered as a possibility versus systemic therapy and/or radiation therapy.

### 3.1. Surgical Considerations

Solitary brain metastatic lesion from the lung could be treated by surgical resection with favorable outcome in nonsmall cell lung cancer patients [14]. For isolated metastatic cancer to the thyroid gland, the surgery should be performed in order to avoid the potential morbidity associated with tumor recurrence in the neck, though the prognosis remains poor, for the majority of the cases and it does not contribute to prolonging patients' life [14, 15].

Limited data to date is present in cases with lung cancer lesions and solitary thyroid lesion to recommend surgical resection in these patients with metastatic disease in the thyroid. Surgery remains a consideration if the patient is a surgical candidate with solitary metastatic lesion in the thyroid especially with tracheal or esophageal invasion [1, 16].

In one limited retrospective series, patients with a solitary metastatic thyroid lesion who underwent surgical resection had an overall survival of 34 months versus 25 months for nonsurgical [2]. Surgical resection may include a total thyroidectomy or an isthmolobectomy depending on which preserve the thyroid endocrine function and on the surgical evaluation of the thyroid lesions [2, 3, 7, 8]. Lobo-isthmectomy can be considered to protect the thyroid endocrine functions.

### 3.2. Systemic Therapy

In case of polymetastatic cancer, systemic treatment with chemotherapy or targeted therapy is the standard of care. Radioactive iodine has no place in the treatment of intrathyroid metastases [8]. In our case, the patient was not a surgical candidate given the presence of mediastinal disease in lymph nodes with metastatic lesions so she was treated with systemic therapy.

There was one case report that described a patient with a thyroid metastasis from lung cancer with an epidermal growth factor receptor (EGFR) mutation, and the patient was started on erlotinib and had a marked response in the lung and thyroid mass [17].

### 3.3. Radiation Therapy

External beam irradiation has been described as another best alternative approach for palliation of symptoms due to thyroid metastases [8, 16].

### 3.4. Survival of Patients with Lung Cancer and Metastatic Thyroid Lesions

The actual survival of patients with thyroid metastases is variable and depends on the primary cancer; survival is significantly better if the primary cancer is renal, compared with extrarenal locations [4]. Prolonged survival more than five years has been observed for patients with thyroid metastases who were surgical candidates [1, 7, 8, 10]. In case of multiple metastases, the survival rate at five years is 5% [3].

## 4. Conclusion

For patients with a thyroid mass or even recurrent laryngeal paralysis with a previous history of malignancy, the thyroid metastasis should be considered.

For patients without such a history, the distinction by clinical features, imaging and FNA should be used to distinguish between a primary thyroid cancer and a metastatic disease should also be of consideration due to the significant difference in the therapeutic approach.

For thyroid metastases, isolated thyroidectomy could be considered in case of solitary metastatic involvement. Systemic therapy should be used in case of widely metastatic disease. External beam irradiation of the gland is alternative palliative approach.

There was an unknown correlation with EGFR exon 19, 21 mutation or EML4-ALK gene rearrangement in the literature. Our patient was negative for both and thus could not be treated with targeted therapy as a front line for her metastatic disease.
